# Daily step count and all-cause mortality in a sample of Japanese elderly people: a cohort study

**DOI:** 10.1186/s12889-018-5434-5

**Published:** 2018-04-23

**Authors:** Naofumi Yamamoto, Hideo Miyazaki, Mieko Shimada, Naoki Nakagawa, Susumu S. Sawada, Mamoru Nishimuta, Yasuo Kimura, Ryoko Kawakami, Hiroshi Nagayama, Hidenori Asai, I-Min Lee, Steven N. Blair, Yutaka Yoshitake

**Affiliations:** 1 Ehime University, 3 Bunkyo-cho, Matsuyama, Ehime 790-8577 Japan; 2 Graduate School of Medical and Dental Sciences, Niigata University, 2-5274, Gakkocho-dori,Chuo-ku, Niigata, 951-8514 Japan; 3 Chiba Prefectural University of Health Sciences, 2-10-1, Wakaba, Mihama-ku, Chiba 261-0014 Japan; 4 SANNO University, 1573 Kamikasuya, Isehara, Kanagawa 259-1197 Japan; 5 Waseda University, 2-579-15 Mikajima, Tokorozawa, Saitama 359-1192 Japan; 6 Toyo University, 1-1-1 Izumino, Itakura-machi, Ora-gun, Gunma, 374-0193 Japan; 7 Research Center for Fitness & Health Sciences, 2-8-9-808, Takada, Toyoshima-ku, Tokyo, 171-0033 Japan; 8 Kyushu Otani Junior College, 495-1 Kurakazu, Chikugo, Fukuoka 833-0054 Japan; 9 Division of Preventive Medicine, Brigham and Women’s Hospital and Harvard Medical School, 75 Francis Street, Boston, MA 02115 USA; 10 Department of Epidemiology, Harvard T.H. Chan School of Public Health, 677 huntington Avenue, Boston, MA 02115 USA; 11 Arnold School of Public Health, University of South Carolina, 921 Assembly Street, Columbia, CA 29208 USA; 12 National Institute of Fitness and Sports in Kanoya, 1 Shiromizu-cho, Kanoya, Kagoshima 891-2393 Japan

**Keywords:** Pedometer, Walking, Physical activity, Objective measurement, Follow-up study

## Abstract

**Background:**

This study aimed to examine the relationship between pedometer-assessed daily step count and all-cause mortality in a sample of elderly Japanese people.

**Methods:**

Participants included 419 (228 males and 191 females) physically independent, community-dwelling 71-year-old Japanese people. The number of steps per day was measured by a waist-mounted pedometer for seven consecutive days at baseline. Participants were divided into quartiles based on their average number of steps/day (first quartile, < 4503 steps/day; second quartile, 4503–6110 steps/day; third quartile, 6111–7971 steps/day; fourth quartile, > 7972 steps/day) and were followed up over a mean period of 9.8 years (1999–2010) for mortality.

**Results:**

Seventy-six participants (18.1%) died during the follow-up period. The hazard ratios (adjusted for sex, body mass index, cigarette smoking, alcohol intake, and medication use) for mortality across the quartiles of daily step count (lowest to highest) were 1.00 (reference), 0.81 (95%CI, 0.43–1.54), 1.26 (95%CI, 0.70–2.26), and 0.46 (95%CI, 0.22–0.96) (*P* for trend = 0.149). Participants in the highest quartile had a significantly lower risk of death compared with participants in the lowest quartile.

**Conclusion:**

This study suggested that a high daily step count is associated with a lower risk of all-cause mortality in physically independent Japanese elderly people.

## Background

Previous long-term longitudinal studies have found that elderly individuals (≥65 years) with lower physical activity levels have a higher risk of all-cause mortality, all deaths regardless of cause, than those with higher physical activity levels [1, 2]. These studies also showed a linear dose-response relationship between the two [1, 2]. Since walking represents a major component of daily physical activity in the elderly [3], low levels of walking activity are considered to be a risk factor for all-cause mortality [1]. Most previous studies investigating the relationship between walking activities and all-cause mortality in the elderly have relied on questionnaire methods to measure daily walking activities [1, 4, 5]. However, it is often difficult to clearly remember intermittent physical activities (e.g., incidental walking, as opposed to walking for exercise); thus, measurement bias due to the inaccurate recollection of events and incorrect answers, especially from the elderly, is a major issue [6]. Therefore, it is preferable to use objective techniques to accurately evaluate the relationship between daily walking activities and all-cause mortality in the elderly [7].

The pedometer is a widely recognized, useful, and objective means of measuring walking activities in daily life and is valid and reliable [8]. Since it is convenient, its use is also recommended in epidemiological studies [8]. However, to the best of our knowledge, the only previous study that has investigated the relationship between daily step count and all-cause mortality is a report by Dwyer et al. [9] that examined an Australian population. In addition, only a few studies have examined the relationship between objectively measured physical activity and mortality in elderly people [9–11]. No study has investigated Japanese populations, which have different genetic backgrounds and lifestyles than those of Westerners. A difference in life expectancy depending on race, gender and/or lifestyles has been suggested by epidemiological data [12]. Furthermore, many studies have shown that gender [13] and lifestyles [14] are associated with all-cause mortality in elderly people. Therefore, the aim of the present study was to examine the confounder-adjusted relationship between daily step count, measured using a pedometer, and all-cause mortality risk in elderly Japanese individuals.

## Methods

### Participants

**Fig. 1:**
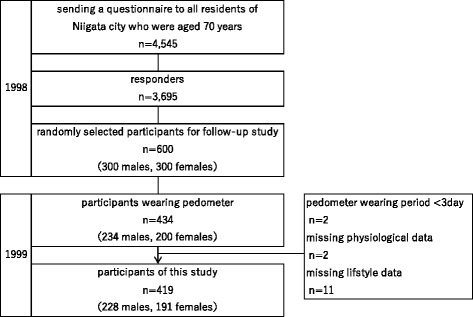
Flow diagram of participants selection in this study

This analysis is part of a long-term longitudinal study on the relationship between various environmental factors and health outcomes in elderly people living in Niigata City, Japan [15, 16]. We sent a questionnaire to all residents (*n* = 4542) of Niigata City who were aged 70 years (born between April 1927 and March 1928) in 1998 to invite them to participate in a health-related long-term longitudinal study to assess levels of physical independence. From the 3695 (81.4%) responders, we randomly selected 300 males and 300 females, totaling 600 people for the baseline survey. None of these participants were hospitalized or institutionalized individuals. The details of this study were described in a prior report [15, 16]. In 1999 (at age 71), the physical activity of 434 of the 600 participants (234 men and 200 women) was measured using a pedometer. Most individuals who refused measurement cited the burden of wearing the device and keeping records as their reason. In this study, 419 participants (228 men and 191 women) were followed up until 2010, except for two whose step activity data did not satisfy the period specified in the criteria (3 days) [17], two whose physical measurement values were not obtained, and 11 whose questionnaires were missing answers to some items (Fig. 1).

We have provided complete details about the purpose of this study, procedures, and risk of measurements and have obtained signed written consent for participation from all participants. This study was approved by the Ethics Committee of the Faculty of Dentistry, Niigata University.

### Physical activity assessment

A spring-levered pedometer (EC-100S, YAMASA, Tokyo, Japan) was used to measure steps as an objective index of physical activity. A pedometer that satisfied the mechanical precision prescribed by Japanese Industrial Standards was used. To meet the specifications of Japanese Industrial Standards, a pedometer should display the number of steps within ±3% after 1000 trials on vibration testing equipment. A prior study reported that the steps per day measured by a pedometer (EC-200, YAMASA, Tokyo, Japan) with the same specifications as the one used in this study showed a significant correlation (*r* = 0.962) with the steps per day measured by the Lifecorder (Kenz, Aich, Japan) [18], which has very high measurement accuracy [19–21]. Participants were fully instructed on the operation of a pedometer, which they were then given to use. They were instructed to wear the pedometer at the waist, at a point extending from the left mid-thigh, continuously for 1 week while they were awake, except for while they were bathing. Participants were also instructed to record steps per day and compliance (whether or not to wear the pedometer during all waking hours) of each day in a log. Steps were measured from June to July 1999. After collecting the logs, the mean daily values were calculated and used for analyses after confirming that no data were missing for three or more days [17].

### Other measurements

Before the start of the follow-up period (1999), baseline data were collected for height, weight, body mass index (BMI), percentage of body fat, alcohol intake, cigarette smoking, and medication use, which are known or potential confounders. BMI was calculated based on height and weight. Percentage of body fat was measured in the early morning after fasting for at least 10 h using a body-fat scale (TBF-310, TANITA, TBF-310, Tokyo, Japan) based on a bioelectrical impedance method. Alcohol intake, cigarette smoking, and medication use were confirmed with a self-administered questionnaire.

### Mortality

Survival of participants was monitored from July 1999 to July 2010. A health survey was completed by the participants at their convenience once a year in July. The participants who did not participate in the health survey were visited at their homes, or they (or a family member) were contacted by phone to verify their survival status. When death was reported, the date of death was determined. When a participant was lost to follow-up due to moving from the target area or other reasons, the data were cut off as of the date of loss to follow-up.

### Statistical analysis

A t-test and χ^2^-test were applied to assess the differences between men and women. Based on the data distribution of steps per day, participants were divided into quartiles. A one-way ANOVA with post hoc Bonferroni test and χ^2^-test was applied to assess the differences in the confounders between the quartiles. To examine the relationships with steps per day, hazard ratios for all-cause mortality and 95% confidence intervals (CI) were calculated for other quartiles using a Cox proportional hazard model based on the first quartile (fewest steps per day). Sex, BMI (continuous variable), cigarette smoking (never-smokers, past smokers, current smokers), alcohol intake (non, 1–2 times/week, 3–5 times/week, 6–7 times/week), and medication use (yes, no) were incorporated into the model to adjust for known or suspected confounders. The relationships between steps per day and all-cause mortality were also confirmed using a spline curve.

The proportionality assumption of the models was tested using a log-minus-log plot; no evidence of violation was found. Sensitivity analysis was performed by excluding all-cause deaths within 3 years after the start of follow-up to eliminate the effect of possible preexisting disease at baseline. The relationships between steps per day and all-cause mortality were also examined in a model incorporating percentage of body fat instead of BMI. Moreover, the interaction between steps per day and sex and all-cause mortality was examined.

Stata version 14.1 (Stata Corp, College Station, TX, USA) was used for all statistical analysis, and a significance level was set as a P less than 5% in a 2-tailed test.

## Results

The mean (range) follow-up period was 9.8 years (1–11 years), and 75.7% of the participants were followed for 11 years. During the follow-up period, 76 participants (60 men and 16 women) died, and 26 (6.2%) were lost to follow-up. Of the 228 men, 226 measured steps for 7 days, and two measured steps for 6 days. All women in the study measured steps for 7 days.

The baseline characteristics overall and according to sex are shown in Table 1. No significant difference in steps per day existed between men and women. Cigarette smoking and alcohol intake rates were significantly lower in women. The baseline characteristics of participants according to quartiles (steps per day) are shown in Table 2. BMI in the first quartile was significantly higher than that in the fourth quartile. No definite trend was observed for smoking, drinking or medication use.

**Table 1:** Baseline characteristics of participants

	All	Males	Females	*p*
Number of participants	419	228	191	
Steps/day (steps)	6470 (2732)	6605 (2882)	6308 (2540)	0.294
Height (cm)	156.8 (8.2)	162.7 (5.4)	149.8 (4.7)	< 0.001
Weight (kg)	55.6 (8.8)	59.2 (8.4)	51.4 (7.4)	< 0.001
Body mass index (kg·m^−2^)	22.6 (2.9)	22.3 (2.8)	22.9 (3.1)	0.036
Percentage of Body fat (%)	23.8 (7.1)	19.8 (5.1)	28.4 (6.2)	< 0.001
Smokers				< 0.001
Never smoker (%)	47.0	11.0	90.1	
Past smoker (%)	34.6	58.8	5.8	
Current smoker (%)	18.4	30.3	4.2	
Drinkers				< 0.001
Never drinker (%)	52.5	29.8	79.6	
1–2 times/week (%)	9.1	8.3	9.9	
3–5 times/week (%)	9.8	14.0	4.7	
6–7 times/week (%)	28.6	47.8	5.8	
Medication use (%)	62.5	63.6	61.3	0.685

Data represents mean (standard deviation) or %

**Table 2:** Baseline characteristics of participants, according to steps/day levels (quartiles)

	First	Second	Third	Fourth	*p*
	quartile	quartile	quartile	quartile	
Number of participants	105	104	105	105	
Steps/day, range (steps)	< 4503	4503–6110	6111–7971	> 7972	
Steps/day, mean (steps)	3394 (824)	5310 (503)	6924 (539)	10,241 (1822)	< 0.001
Height (cm)	156.7 (8.3)	156.9 (8.4)	156.2 (8.1)	157.4 (8.1)	0.788
Weight (kg)	57.3 (9.1)	55.3 (9.1)	55.0 (8.2)	55.0 (8.8)	0.202
Body mass index (kg·m^−2^)	23.3 (3.2)*	22.4 (2.9)	22.5 (3.0)	22.1 (2.6)	0.036
Percentage of Body fat (%)	25.0 (7.7)	23.8 (7.1)	23.9 (7.2)	22.3 (6.1)	0.062
Female (%)	45.7	46.2	50.5	40.0	0.504
Smokers (%)	21.9	23.1	13.3	15.2	0.117
Drinkers (%)	39.0	35.6	35.2	43.8	0.594
Medication use (%)	61.0	69.2	61.9	58.1	0.391

Data represents mean (standard deviation) or %

Smokers, percentage of current cigarette smokers

Drinkers, percentage of current alcohol drinkers (3 days or more per week)

*p < 0.05 vs Fourth quartile

The relationships between known or suspected confounders and all-cause mortality are shown in Table 3. The hazard ratio was higher in men compared to women. No significant relationships between BMI, percentage body fat, smoking, and medication use groups were observed. The hazard ratios of participants who drank 6–7 times per week were significantly lower than of those participants who never drank.

**Table 3:** Adjusted hazard ratios for all-cause mortality by known or suspected confounders

	Participants	Hazard ratio	95%CI
Sex^*a*^			
Female	191	1.00 (reference)	
Male	228	2.60	1.12–6.07
Body mass index^*b*^			
First quartile 15.1–20.5 kg·m^−2^	108	1.00 (reference)	
Second quartile 20.6–22.4 kg·m^− 2^	105	0.60	0.31–1.16
Third quartile 22.5–24.6 kg·m^−2^	104	0.78	0.41–1.51
Fourth quartile 24.7–31.8 kg·m^−2^	102	1.04	0.57–1.88
Percentage of Body fat^*b*^			
First quartile 7.7–18.9%	107	1.00 (reference)	
Second quartile 19.0–23.2%	106	0.91	0.50–1.66
Third quartile 23.3–28.6%	102	1.16	0.62–2.17
Fourth quartile 28.7–49.7%	104	0.90	0.38–2.13
Cigarette smoking^*c*^			
Never smoker	197	1.00 (reference)	
Past smoker	145	1.65	0.73–3.76
Current smoker	77	2.12	0.90–4.98
Alcohol intake^*d*^			
Never drinker	220	1.00 (reference)	
1–2 times/week	38	1.03	0.47–2.28
3–5 times/week	41	1.23	0.63–2.41
6–7 times/week	120	0.55	0.30–0.99
Medication use^*e*^			
No	157	1.00 (reference)	
Yes	262	1.60	0.96–2.68

^a^Adjusted for steps/day (continuous variable), body mass index (continuous variable), cigarette smoking (never-smokers, past smokers, current smokers), alcohol intake (non, 1–2 times/week, 3–5 times/week, 6–7 times/week), and medication use (yes, no)

^b^Adjusted for steps/day (continuous variable), sex, cigarette smoking (never-smokers, past smokers, current smokers), alcohol intake (non, 1–2 times/week, 3–5 times/week, 6–7 times/week), and medication use (yes, no)

^c^Adjusted for steps/day (continuous variable), sex, body mass index (continuous variable), alcohol intake (non, 1–2 times/week, 3–5 times/week, 6–7 times/week), and medication use (yes, no)

^d^Adjusted for steps/day (continuous variable), sex, body mass index (continuous variable), cigarette smoking (never-smokers, past smokers, current smokers), and medication use (yes, no)

^e^Adjusted for steps/day (continuous variable), sex, body mass index (continuous variable), cigarette smoking (never-smokers, past smokers, current smokers), and alcohol intake (non, 1–2 times/week, 3–5 times/week, 6–7 times/week)

The sex-adjusted and multivariable-adjusted hazard ratios for all-cause mortality according to quartiles (steps per day) are indicated in Table 4. There was no linear relationship between steps per day and all-cause mortality (*P* for trend = 0.149); however, the hazard ratio of the fourth quartile was significantly lower than that of the first quartile (HR = 0.46). While the same trend was observed in men, the 95% CI was significantly wider, and no definite trend in the hazard ratio was observed in women, probably due to fewer deaths in women than in men.

**Table 4:** Adjusted hazard ratios for all-cause mortality according to steps/day levels (quartiles)

	First quartile	Second quartile	Third quartile	Fourth quartile	P for trend
All participants					
Number of participants	105	104	105	105	
Person-years of follow-up	1019	1007	983	1099	
Number of decedents	22	18	25	11	
Death rate per 10,000 person-years	216	179	254	100	
Sex adjusted HR (95%CI)	1.00 (reference)	0.84 (0.45–1.56)	1.23 (0.69–2.19)	0.42 (0.20–0.87)	0.080
Multivariable adjusted HR^*a*^(95%CI)	1.00 (reference)	0.81 (0.43–1.54)	1.26 (0.70–2.26)	0.46 (0.22–0.96)	0.149
Male					
Number of participants	57	56	52	63	
Person-years of follow-up	526	514	476	666	
Number of decedents	17	16	19	8	
Death rate per 10,000 person-years	323	311	399	120	
Crude HR (95%CI)	1.00 (reference)	0.97 (0.49–1.92)	1.25 (0.65–2.40)	0.37 (0.16–0.86)	0.056
Multivariable adjusted HR^*b*^ (95%CI)	1.00 (reference)	1.00 (0.49–2.01)	1.34 (0.68–2.62)	0.44 (0.19–1.03)	0.165
Female					
Number of participants	48	48	53	42	
Person-years of follow-up	493	493	507	433	
Number of decedents	5	2	6	3	
Death rate per 10,000 person-years	101	41	118	69	
Crude HR (95%CI)	1.00 (reference)	0.40 (0.08–2.07)	1.17 (0.36–3.84)	0.68 (0.16–2.86)	0.945
Multivariable adjusted HR^*b*^ (95%CI)	1.00 (reference)	0.36 (0.07–1.93)	1.14 (0.35–3.76)	0.66 (0.15–3.01)	0.985

*HR* hazard ratio, *CI* confidence interval

^*a*^ Adjusted for sex, body mass index (continuous variable), cigarette smoking (never-smokers, past smokers, current smokers), alcohol intake (non, 1–2 times/week, 3–5 times/week, 6–7 times/week), and medication use (yes, no)

^*b*^ Adjusted for body mass index (continuous variable), cigarette smoking (never-smokers, past smokers, current smokers), alcohol intake (non, 1–2 times/week, 3–5 times/week, 6–7 times/week), and medication use (yes, no)

**Fig. 2:**
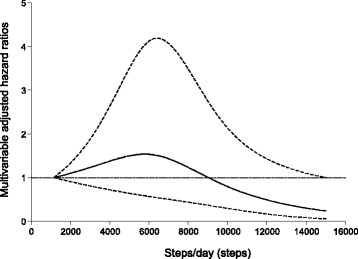
Restricted cubic spline regression for the association between steps/day levels and all-cause mortality. Data were fitted using a Cox proportional hazards model after adjusting for sex, body mass index (continuous variable), cigarette smoking (never-smokers, past smokers, current smokers), alcohol intake (non, 1–2 times/week, 3–5 times/week, 6–7 times/week), and medication use (yes, no). The solid line represents hazard ratios and the dashed line represents 95% confidence intervals. Knots were placed at the 10th, 50th, and 90th percentiles (3274, 6111, 10,224 steps/day) of daily step levels and using 1160 steps/day as a reference point

The relationships between steps per day and all-cause mortality using a spline curve are shown in Fig. 2. Although the 95% CI contained a hazard ratio of 1.00, taking approximately 9000 or more steps/day was associated with a hazard ratio lower than the reference (1.00).

A sensitivity analysis was performed to exclude participants who died within 3 years after the start of follow-up to eliminate the effects of possible preexisting disease at baseline on the relationships between steps per day and all-cause mortality. The results showed a trend similar to that for the results that included participants who died within 3 years after the start of follow-up (*P* for trend = 0.401). The hazard ratios for death across the quartiles of daily step count (lowest to highest) were 1.00 (reference), 0.80 (95%CI, 0.38–1.66), 1.46 (95%CI, 0.75–2.81), and 0.54 (95%CI, 0.24–1.19). The results of models using either percentage of body fat or BMI were similar (*P* for trend = 0.134). No significant interaction was detected in examination of the associations between steps per day, sex, and all-cause mortality (*P* for interaction = 0.442). Furthermore, we performed analysis with very low level of steps/day (< 2250 steps/day) as a reference. The hazard ratios for death of the first, second, third, and fourth quartiles with respect to the reference were 0.50 (95%CI, 0.16–1.51), 0.44 (95%CI, 0.14–1.36), 0.66 (95%CI, 0.23–2.05), and 0.25 (95%CI, 0.08–0.82), respectively (*P* for trend = 0.112). According to the results of the model using continuous steps per day as exposure, the hazard ratio for death was 0.93 (95%CI, 0.85–1.01) per 1000 steps (*P* for linearity = 0.078).

## Discussion

This is the first study to examine the relationship between objectively measured physical activity and mortality in elderly Japanese individuals. Using step count data allows the results to be translated easily to the general population. This study suggested that a high daily step count is associated with a lower risk of all-cause mortality in physically independent Japanese elderly people.

In the current study, the mean daily step count was 6605 for men and 6308 for women. Tudor-Locke et al. [22] reported that the total daily step count of the typical U.S. elderly person is 2000–9000. In addition, the National Health and Nutrition Survey, a large-scale survey conducted using Japanese participants, reported that the mean daily step count of those aged 65–74 years in 2014 was 6562 for men and 5594 for women in Japan [23]. The mean daily step count of the participants in this study were approximately the same as the levels in the precious studies.

In the present study, no statistically significant linear relationship was found between daily step count and all-cause mortality. Recently, the relationship between physical activity and all-cause mortality in the elderly has been investigated using objective methods of measuring physical activity (e.g., the doubly labeled water method [10], pedometers [9], and accelerometers [11]). Accordingly, a linear dose–response relationship has been shown between the two parameters. The results of our study differed from those obtained from the studies by Manini et al. [10], Ensrud et al. [11], and Dwyer et al. [9]. The reason for this discrepancy is considered to be the effect of small sample size and selection bias. One hundred and sixty-six out of the 600 subjects declined to cooperate in having their number of steps measured. It has been suggested that individuals who do not consent to activity monitor-based measurement are likely participants with lower physical activity levels [24]. It has also been reported that noncooperators to physical fitness measurement have a higher risk of death than cooperators [25]. Thus, it is likely that the selection process of this study selectively excluded participants with fewer steps and with a higher risk of death. This reason may explain why we did not observe a linear relationship between daily step count and all-cause mortality. However, our spline curve showed that taking approximately 9000 steps per day or more was associated with a hazard ratio lower than the reference (Fig. 2). In addition, we observed a negative dose-response relationship for participants who took more than 9000 steps/day. Furthermore, the fourth quartile demonstrated a significantly lower hazard ratio than the first quartile (Table 4). The ratio of walking activities to total physical activities in daily life increases with aging [3]. Therefore, daily step count in the elderly can be considered to be a reflection of the level of physical activity in their daily lives. A number of studies have reported that the maintenance of higher physical activity in the elderly lowers the risk of various chronic diseases and geriatric syndromes as well as the risk of death [7]. Thus, this study investigated elderly Japanese people using an objective physical activity measurement, and in line with previous studies, it suggests the likelihood that higher physical activity levels have preventive effects on all-cause mortality.

The American College of Sports Medicine [26] and Global Recommendations on Physical Activity for Health published by the WHO [27] recommend moderate physical activity for approximately 30 min per day as a minimum physical activity target value for the elderly to maintain and promote their health. Based on a review of previous studies, Tudor-Locke et al. [22] equated the value recommended by these physical activity guidelines (moderate physical activity for 30 min or more daily) to a total of 7000–10,000 daily steps. The reference value for daily step count obtained from the fourth quartile in this study (> 7972 steps per day) was roughly equivalent to the value described above. Moreover, the Nakanojo study, which cross-sectionally examined the relationship between daily step count and various health measures in elderly Japanese people, reported that the more daily steps subjects had above 8000 steps/day, the better physical health conditions they exhibited (e.g., metabolic fitness and physical fitness, associated with leading causes of death in Japan—cancer and cardiovascular diseases) [28]. Therefore, a significantly lower hazard ratio for death in the fourth quartile may be observed in this study.

The strength of this study is its objective measurement of physical activity. Furthermore, almost all participants wore the pedometer continuously for 1 week. Therefore, we believe that the step count of the participants accurately reflected their daily walking activities. This study also has some limitations. First, we failed to secure a sufficiently large sample size to evaluate the relationship between daily step count and all-cause mortality by sex. The present study obtained only a small number of deaths of women during the follow-up period. We did not find a significant interaction between daily step count and sex concerning all-cause mortality (*P* for interaction = 0.442). Similarly, Dwyer et al. [9] also did not find sex-dependent relationships between daily step count and all-cause mortality. Therefore, we assume that a high daily step count is associated with a lower all-cause mortality risk in both men and women. However, previous reports have also found that among the elderly, men tend to have more steps than women [23]. Moreover, since elderly Japanese women traditionally engage in housekeeping for longer hours than men, the ratio of low-intensity physical activity time to the total physical activity time is higher in women than in men [28]. Therefore, walking intensities may be different between men and women, even if these populations have the same number of steps. Thus, it is considered necessary to investigate how the total daily step count based on step quality (e.g., fast or slow steps) is associated with the all-cause mortality risk by sex in elderly Japanese populations using larger sample sizes. Second, the measurement of the daily step count was performed only once at baseline. However, in a previous study that examined the same population as that in the present study reported high levels of tracking (maintenance of participants’ relative ranks within the population over time) concerning the daily step count in the elderly [29]. Thus, although the measurement was made only at baseline in the present study, it seems reasonable that the relative physical activity levels of individuals were stable during the follow-up period. The final limitation is that the study subjects were those who consented to pedometer-based measurements, representing a physically independent, healthy elderly population rather than a sample population representing the elderly in general. Therefore, the results of this study may not necessarily be applied to the general elderly population. Admitting these limitations, to the best of our knowledge, we believe that this is the first study to examine the relationship between daily step count and all-cause mortality in elderly Japanese individuals. This study provides important data to help enhance public health-related significance in measuring daily step counts in the elderly.

## Conclusions

This study suggested that a high daily step count is associated with a lower risk of all-cause mortality in physically independent Japanese elderly people.

## Abbreviations

BMI: Body mass index
CI: Confidence interval
HR: Hazard ratio
WHO: World Health Organization
